# New Onset Insomnia in a Pediatric Patient: A Case of Anti-NMDA Receptor Encephalitis

**DOI:** 10.1155/2017/4083785

**Published:** 2017-07-09

**Authors:** Tamar N. Goldberg, Michael F. Cellucci

**Affiliations:** ^1^Department of Pediatrics, Sidney Kimmel Medical College/Alfred I. duPont Hospital for Children Pediatric Residency Program, Wilmington, DE, USA; ^2^Department of Neurology, Boston Children's Hospital, Child Neurology and Neurodevelopmental Disabilities Residency Program, Boston, MA, USA; ^3^Division of Diagnostic Referral, Department of Pediatrics, Nemours/Alfred I. duPont Hospital for Children, Wilmington, DE, USA

## Abstract

Anti-NMDAR encephalitis is becoming more widely recognized as a cause of encephalopathy in both adults and children. Certain clinical features such as mood lability, movement disorders, speech dysfunction, seizures, and autonomic instability in a pediatric patient should prompt immediate concern and evaluation for autoimmune encephalitis among providers. We present the case of a pediatric patient with anti-NMDAR encephalitis in which the symptom prompting medical evaluation was insomnia. Insomnia has not previously been emphasized in the literature as a presenting feature of this disease in children and has a broad differential. Recognition of the symptoms of anti-NMDAR encephalitis and its variable presentation are key to early diagnosis and prompt initiation of treatment which may help to improve outcomes.

## 1. Introduction

Anti-NMDAR encephalitis is becoming increasingly recognized as a cause of acute and subacute encephalopathy in both adults and children [1]. Although first described in young women as a disorder associated with ovarian teratomas [2], pediatric cases may represent 40% of all cases [3]. Anti-NMDAR encephalitis presents as a multistage illness with most patients experiencing a viral-like prodromal illness followed by psychiatric symptoms including changes in behavior and speech, emotional lability, and irritability or psychosis. The illness progresses to neurologic symptoms with catatonia, mutism, and seizures, followed by dyskinesia and autonomic instability [4–7]. Children typically are brought to medical attention because of subtle changes in mood, behavior, personality, or language regression [3].

Awareness of the disease and recognition of its symptoms are key to the early diagnosis and prompt initiation of treatment which may improve outcomes. With treatment, 75% of patients are estimated to recover completely while 25% may face residual neurodevelopmental impairment or death [4].

We report a case of a pediatric patient with anti-NMDAR encephalitis in which the symptom prompting medical evaluation was insomnia which has not been previously emphasized in the literature as a presenting feature of anti-NMDAR encephalitis.

## 2. Case Presentation

A five-year-old previously healthy bilingual (English and Spanish) boy presented to the ED with his parents due to insomnia for the previous two nights. The family history was negative for neurologic or psychiatric illness. The patient was a typically developing child born after a full term uncomplicated gestation and delivery. He had no prior history of injury, ingestion, psychosocial stressors, change in appetite, involuntary movements, or seizures. One week prior to presentation to the ED, he had been evaluated by his primary care physician after a three-day febrile illness and was diagnosed with a viral infection. Since that time, he had become more irritable and fussy, frequently having outbursts of crying without being able to articulate why he was crying. His father was concerned that he seemed scared during these episodes and was concerned about eye pain.

The patients' initial physical examination revealed normal vital signs, cardiovascular, pulmonary, and abdominal findings. He did not have dysmorphic features or a rash. Neuropsychiatric examination revealed an emotionally labile child with periods of calm and seemingly appropriate bilingual language development and behavior for age with episodes of extreme distress during which he would burst into tears and require physical restraint by family members against combativeness. Examination of the cranial nerves, motor tone, strength, coordination, and gait were normal and there was no sign of meningismus on physical examination.

Initial differential for this clinical presentation was broad including insomnia secondary to pain from corneal abrasion or other unidentified traumatic or infectious source, traumatic brain injury, thyrotoxicosis, developmental disorder with regression, encephalopathy due to acute intoxication, acute psychosis, postinfectious or parainfectious encephalitis, and autoimmune encephalopathy. Screening in the ED with urine drug screen, serum acetaminophen and salicylate levels, complete blood count, complete metabolic panel, thyroid function tests, inflammatory markers, urinalysis, head CT scan, and EKG all revealed normal results. PCR respiratory panel (nasal swab) was negative for frequently tested viral and bacterial pathogens including negative for mycoplasma pneumoniae. The patient was admitted to the general pediatrics service with support from consulting services for further evaluation and management given his altered mental status and concern for acute encephalopathy.

## 3. Hospital Course

After admission, the five-year-old boy continued to have persistent insomnia with intermittent episodes of extreme agitation refractory to several medical therapies including melatonin, diphenhydramine, zolpidem, and lorazepam. A sedated brain MRI and LP were performed on Hospital Day One. No demyelination or other intracranial abnormalities were visualized on MRI. LP revealed a normal opening pressure, white blood cells 17/mm^3^ (90% lymphocytes, 10% monocytes), normal protein level, and normal glucose. Spinal fluid analysis for evaluation of various autoimmune antibodies and infectious pathogens was performed. Laboratory testing was notable for positive EBV VCA IgM and IgG serum antibodies.

**Figure 1:**
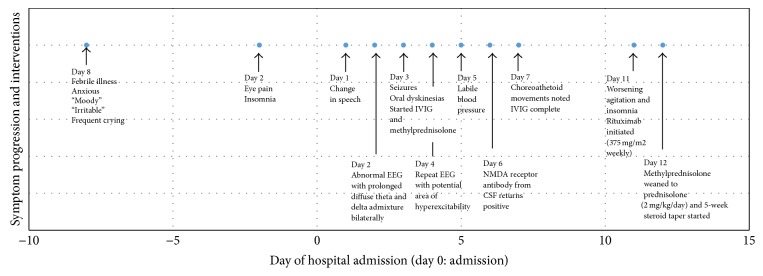
Symptom progression and related interventions during hospitalization.

**Figure 2:**
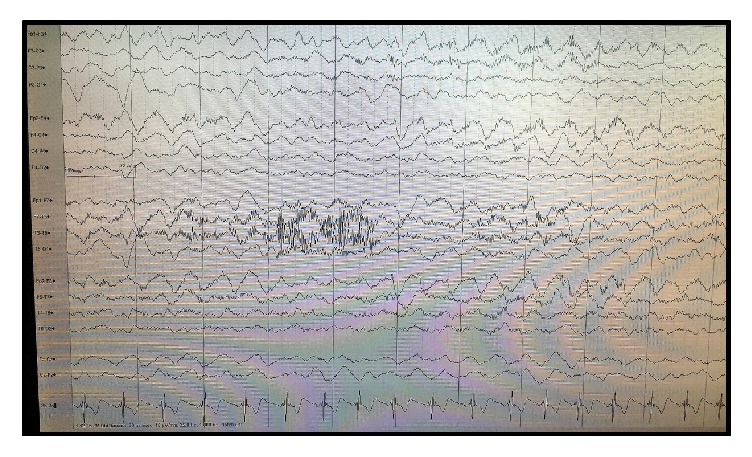
Initial electroencephalogram.

Patient had further decline in his initial days of hospitalization with episodic agitation, insomnia, psychomotor slowing with abnormal posturing, oral-lingual-facial dyskinesia, and progressive aphasia (Figure 1). The EEG was abnormal due to prolonged diffuse theta and delta admixture bilaterally but more prominent on the left suggestive of moderate diffuse cerebral encephalopathy (Figure 2). No clear epileptiform discharges were visualized on the initial EEG. Clonidine and quetiapine were initiated to treat the patient's agitated behavior and insomnia.

The patient had a complex partial seizure with secondary generalization on Hospital Day Three with tachycardia, eye and head deviation, lack of responsiveness, and left upper extremity rhythmic jerking. He was prescribed levetiracetam and later his antiepileptic medication was changed to valproic acid. Repeat EEG was again notable for intermixed delta and theta frequencies of polymorphic morphology with frequent irregular polymorphic delta activity in the bifrontal regions and with hyperexcitability in the right frontal region suggestive of research seizure activity.

On Hospital Day Three, he began empiric treatment with nine days of 10 mg/kg/day intravenous methylprednisolone and a five-day course of 400 mg/kg/day intravenous immunoglobulin given high clinical suspicion for autoimmune encephalitis based on his progressive multistage illness and clinical features (Figure 1). Bacterial and viral CSF studies returned negative. Autoimmune and paraneoplastic laboratory evaluation on CSF and serum were diagnostic for anti-NMDAR encephalitis with positive serum anti-NMDA receptor antibodies with a titer of 1 : 400 and positive CSF anti-NMDA receptor antibodies with a titer of 1 : 4.

Treatment with once weekly rituximab 375 mg/m^2^ for four weeks was started on Hospital Day Ten given persistence and progression of symptoms. Twice daily mycophenolate was started and a five-week steroid taper was initiated. Clinical course was further complicated by dysautonomia and loss of purposeful motor movement. The patient continued to have limited receptive and expressive speech abilities with agitation and mood lability and remained hospitalized for ongoing rehabilitation and management. He was discharged home on Hospital Day 68 and has had close outpatient follow-up with multiple subspecialties. Medications to manage disruptive behaviors (clonidine and quetiapine) have since been stopped. He has returned to school and remains seizure-free on valproic acid. He is maintained on twice daily mycophenolate and has had no recurrence of dyskinesia or sleep disturbance.

## 4. Discussion

Sleep problems are a common pediatric neuropsychiatric complaint and occur in up to one-third of all children. Although most of the underlying processes are benign and self-limited, some serious illnesses can present with insomnia. Pediatric insomnia is defined as ongoing difficulty with sleep initiation, duration of sleep, or quality of sleep despite the opportunity for adequate sleep that results in daytime functional impairment or psychological distress [20].

There is likely a complex interplay between predisposing factors, primary medical conditions, pain, medications, and psychosocial, behavioral, and environmental factors that contribute to insomnia and its association with different medical, psychiatric, and neurologic disorders [8, 9, 21, 25]. The broad differential diagnosis for insomnia in children is reviewed in Table 1. Numerous pathophysiological mechanisms have been implicated in sleep-wake cycle disturbances in medical illnesses. For example, increased levels of proinflammatory cytokines [10], parasympathetic and sympathetic dysregulation, hypothalamic-pituitary-adrenal axis dysfunction, disruption in sleep architecture, and alterations in hormones and neurotransmitters such as melatonin, serotonin, norepinephrine, and dopamine may all play a critical role in the regulation of sleep [11]. In some medical conditions, sleep-wake disruptions may be part of a cluster of symptoms that complicate the diagnosis and management of the patient and may initially be overlooked due to urgent concerns for diagnosis and treatment of primary medical conditions.

Multiple studies have reported sleep-wake cycle disturbances and insomnia as characteristic symptoms in anti-NMDAR encephalitis [7] and while they have been described previously as the presenting feature in adults [16, 17], insomnia in children with anti-NMDAR encephalitis is typically described later in the clinical course [1, 26–29] or has not been mentioned as a feature of the illness [30–32]. Other studies suggest that children are more likely to present with predominantly neurologic symptoms (seizures, abnormal movements, and focal neurologic deficits) than with psychiatric symptoms [26].

Desena et al. (2014) describe a two-year-old girl with anti-NMDAR encephalitis who presented with several weeks of behavioral outbursts and sleep disturbances although the description suggests that it was the progressive behavioral decline in conjunction with agitation and aggression that prompted medical evaluation which is different than the case of our patient in which insomnia was the most concerning and salient feature at onset [33]. Similarly, Pavone et al. (2017) describe a five-year-old girl with anti-NMDAR encephalitis who presented with several weeks of progressive psychiatric and neurologic symptoms starting with unjustified crying, refusal to go to school, difficulty in swallowing, and delayed writing skills which progressed to insomnia with night time awakenings, personality changes, dystonia, and hand and orofacial dyskinesias prior to admission. Although disturbances in sleep-wake cycle are mentioned as part of this patient's clinical symptomatology, the characteristics or management of the insomnia is not a focus of the case report [18].

During our patient's initial evaluation and first days in the hospital, his abrupt mental status changes from extreme agitation with crying and screaming to a relatively calm demeanor inevitably complicated assessments as one physician would find the patient encephalopathic while evaluation a few minutes later by another physician would find him with seemingly developmentally appropriate behavior for a five-year-old in an unfamiliar environment. This phenomenon has been described in other cases of pediatric anti-NMDAR encephalitis as “light switch” mental status changes [33]. Our patient also went on to develop oral-lingual-facial dyskinesia which is classic for anti-NMDAR encephalitis [34]. Although such movements could be confused with tardive dyskinesia occurring as a side effect from antipsychotic medication, he had just recently been started on these antipsychotic medications making movement disorder secondary to progression of his underlying encephalitis much more likely.

Presentation of anti-NMDAR encephalitis has many overlapping features with other conditions including but not limited to viral encephalitis, acute psychosis, and drug-induced psychosis. Diagnosis of anti-NMDAR encephalitis continues to rise and in some studies, anti-NMDAR encephalitis has been found to occur more frequently than encephalitis due to viral pathogens. While certain types of viral encephalitis may more consistently present with fever, rash, and headache, certain clinical features of early presentation should be recognized by clinicians as classic for autoimmune encephalitis. Studies report that symptoms including mood lability, insomnia, agitation, movement disorders, speech dysfunction, and seizures are reported in more cases of anti-NMDAR encephalitis than in encephalitis due to isolated viral etiology [35, 36]. These symptoms in concurrence with rapid changes in mental status, dyskinesia, or dysautonomia should prompt immediate concern for anti-NMDAR encephalitis, especially without prior neuropsychiatric history [7, 37]. While the multistage progression of anti-NMDAR encephalitis has been well described in the literature [4], it is important to acknowledge that each stage of illness will have unique features depending on the case. With additional research, insomnia may become more widely recognized as a presenting symptom to prompt early medical evaluation for other cases of anti-NMDAR encephalitis just as it was for our patient.

## Figures and Tables

**Table 1:** Differential diagnosis for insomnia in children.

*Behavioral insomnia of childhood [8]*
(i) Inconsistent bedtime routine
(ii) Inadequate limit setting
(iii) Use of electronic device
(iv) Parental discord
(v) Emotional, physical, and sexual abuse
*Primary sleep disorders [8, 9] *
(i) Obstructive sleep apnea
(ii) Circadian rhythm sleep wake disorders
(iii) Parasomnias
(iv) Restless leg syndrome
(v) Periodic limb movements of sleep
(vi) Bruxism
*Medical conditions [9–14]*
(i) Allergic rhinitis, atopic dermatitis, otitis media, asthma, chronic cough, and cystic fibrosis
(ii) Colic, gastroesophageal reflux, inflammatory bowel disease, and irritable bowel syndrome
(iii) Malignancy and sickle cell anemia
(iv) Congenital heart disease and cardiac arrhythmias
(v) Juvenile idiopathic arthritis, systemic lupus erythematosus, diabetes, and thyroid disease
(vi) Chronic pain and fibromyalgia
*Acquired central nervous system disorders [7, 15–19]*
(i) Concussion and postconcussive syndrome
(ii) Traumatic brain injury
(iii) Viral, bacterial, or autoimmune meningoencephalitis
(iv) Increased intracranial pressure
(v) Stroke
*Neurologic disorders [9, 11, 20–23]*
(i) Neurodegenerative disease and static encephalopathy
(ii) Autism spectrum disorder
(iii) Cerebral palsy
(iv) Intellectual disability
(v) Visual and/or hearing impairment
(vi) Migraine headaches, tension headaches, and chronic daily headache
(vii) Idiopathic intracranial hypertension
(viii) Epilepsy
(ix) Multiple sclerosis and acute disseminated encephalomyelitis
(x) Myasthenia gravis
*Psychiatric disorders [8, 9, 11, 24]*
(i) Major depressive disorder
(ii) Anxiety disorders
(iii) Posttraumatic stress disorders
(iv) Attention deficit hyperactivity disorder
(v) Bipolar affective disorder
(vi) Schizophrenia
(vii) Psychosis
*Medication-induced insomnia [25]*
(i) Acute intoxication
(ii) Alcohol, tobacco, or illicit drug use
(iii) Caffeine
(iv) Diet pills
(v) Antibiotics
(vi) Antidepressants
(vii) Antipsychotics
(viii) Alpha 2 agonists
(ix) Beta antagonists
(x) Beta agonists
(xi) Glucocorticoids
(xii) Antihistamines
(xiii) Decongestants

## Abbreviations

Anti-NMDAR encephalitis: Anti-N-methyl-D-aspartate receptor encephalitis
CSF: Cerebral spinal fluid
CT: Computerized tomography
EBV: Epstein-Barr Virus
ED: Emergency department
EEG: Electroencephalogram
EKG: Electrocardiogram
LP: Lumbar puncture
MRI: Magnetic resonance imaging
VCA: Viral capsid antigen.
